# A tool of the trade for a newly identified filovirus: pseudovirus-based system for Dehong virus entry and neutralization

**DOI:** 10.1128/spectrum.01035-25

**Published:** 2025-07-29

**Authors:** Banhi Biswas

**Affiliations:** 1 Department of Biochemistry and Molecular Biology, Johns Hopkins Bloomberg School of Public Health 25802 , Baltimore, Maryland, USA; National Microbiology Laboratory, Winnipeg, Manitoba, Canada

**Keywords:** pseudovirus, Dehong virus, neutralization assay

## Abstract

Pseudovirus-based assays are efficient and accessible platforms for measuring neutralizing antibody titers and screening antiviral drugs against enveloped viruses. They typically employ two types of packaging systems, retroviral-based or vesicular stomatitis virus (VSV)-based systems. These systems have been validated for several high-risk pathogens, including Ebola, Marburg, Dengue and, more recently, SARS-CoV-2, as they offer a safe alternative for studying high-containment viruses. In a recent study (Wu X, Liu F, Li T, Li D, Shen Y, Zhang X, Liu S, Jiang Q, Zhao C, Nie J, Wang Y, Feng B, Liu W, Huang W, Microbiol Spectr, 13:e01557-24, 2025, https://doi.org/10.1128/spectrum.01557-24), Wu and colleagues developed a pseudovirus system and optimized it for neutralization assays targeting the newly identified Dehong virus (DEHV). The authors also utilized this system to detect bioluminescent signals in mouse models. Importantly, using this pseudovirus platform, they confirmed that Niemann-Pick C1 (NPC1) functions as the entry receptor for DEHV, consistent with prior findings using infectious viruses. This work provides valuable insights into the limited understanding of DEHV biology.

## COMMENTARY

Dehong virus (DEHV) is a recently discovered member of the Filoviridae family, which includes highly pathogenic viruses, such as Ebola (EBOV) and Marburg (MARV), both known to cause severe hemorrhagic fevers (1). DEHV was isolated from *Rousettus leschenaultii* bats and exhibits unique genomic features, including the largest genome among known filoviruses. It also displays broad cellular tropism and, like EBOV, MARV, Mengla virus (MLAV), and Lloviu virus (LLOV), it utilizes NPC1 as its entry receptor (2). Given its recent discovery, very little is known about DEHV’s biology or pathogenesis, an important knowledge gap, especially considering that fruit bats, the DEHV reservoir species, often forage in jujube orchards located near human populations (2). While no human infections have been reported across Asia to date, the zoonotic potential remains a concern.

Wu et al. (3) represents the first successful development of DEHV pseudoviruses bearing the virus’s glycoprotein (GP), which were subsequently used to immunize mice and measure neutralizing antibody responses. Pseudoviruses offer a safer and less labor-intensive alternative to working with highly pathogenic viruses, such as Ebola (4) and Marburg (5), which require BSL-4 containment. Lacking the virulent components of the native virus, pseudoviruses are much safer to handle (6). Moreover, pseudovirus-based systems are particularly well-suited for quantifying neutralizing antibody titers, as they are engineered to produce measurable luminescent signals, enabling precise and high-throughput analyses (7, 8). These systems also provide a robust and convenient platform for dissecting viral entry mechanisms across diverse viral glycoproteins (6, 9).

Wu et al. (3) immunized mice with their pseudovirus system and collected serum samples for up to 7 weeks post-immunization for neutralization assays. The study also carefully optimized the neutralization assay conditions, including cell seeding density and time points for neutralization antibody titer measurement. The anti-sera could neutralize DEHV pseudovirus in a neutralization assay in HEK-293T cells. Importantly, the DEHV anti-sera failed to neutralize MARV or MLAV pseudoviruses, which is expected given the ~50% sequence similarity between DEHV and MARV GPs. Moreover, sera from individuals infected with other filoviruses, unrelated RNA viruses, or healthy human controls did not neutralize DEHV pseudoviruses, highlighting the specificity of the assay. Future testing of bat-derived antisera (2) against the DEHV pseudovirus system can further validate the robustness of the system.

Next, using the pseudovirus system, the authors confirmed that NPC1 is essential for DEHV entry, as cells lacking NPC1 did not support infection, thus validating previous findings (2). The pseudovirus platform developed here offers a powerful tool for dissecting the molecular determinants of GP-mediated entry. It could also be adapted for the rapid screening of neutralizing antibodies following spillover events in non-human primates or humans.

Finally, they also injected the DEHV pseudoviruses into various mouse models via intravenous and intraperitoneal routes to monitor viral localization using bioluminescent imaging. Signals were first detected 2 days post-infection, peaked by day 5, and declined by day 8. However, as the system only supports a single round of replication, using this system for organ/tissue tropisms is not optimal.

While the study successfully establishes a pseudovirus system, it is important to note that neutralization outcomes observed with pseudoviruses do not always correlate with those from the authentic virus (10). Hence, direct comparison with live virus assays remains essential. Additionally, in the absence of DEHV GP-specific antibodies, it is currently challenging to confirm the incorporation of DEHV glycoprotein into the pseudoviruses, underscoring the need for further validation of the system. Also, the density and distribution of GP in the pseudovirus system may not be similar to that of the native virus, thus altering neutralization potential and sensitivity (6, 8). Therefore, although pseudovirus-based systems are easy to generate and handle, one must consider these possibilities.
